# Supplementary material for: A Systematic Review of Government-Led Free Caesarean Section Policies in Low- and Middle-Income Countries from 2009 to 2025
Source: Healthcare (Basel). 2025 Oct 4;13(19):2522. doi: 10.3390/healthcare13192522 (PMC12524749; doi:10.3390/healthcare13192522)
Supplement: Supplementary file 1 [file healthcare-13-02522-s001.zip › Figure S1a (CASP), Figure S1b (EPOC-ITS), and Figure S1c (ROBINS-I).pdf]

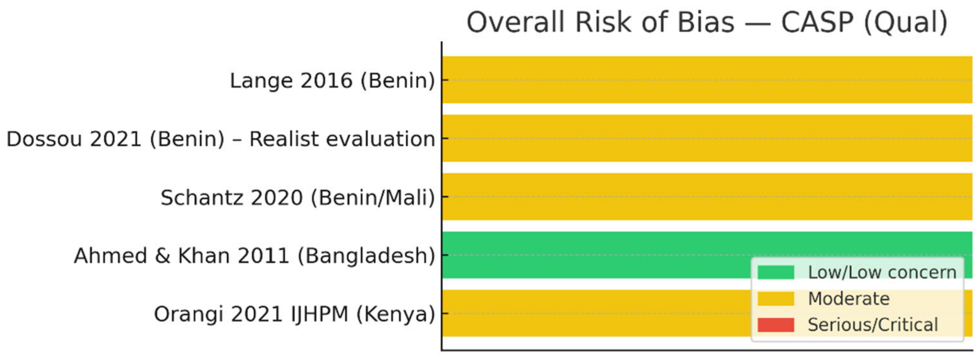


**Figure S1a: CASP Overall Traffic Light**

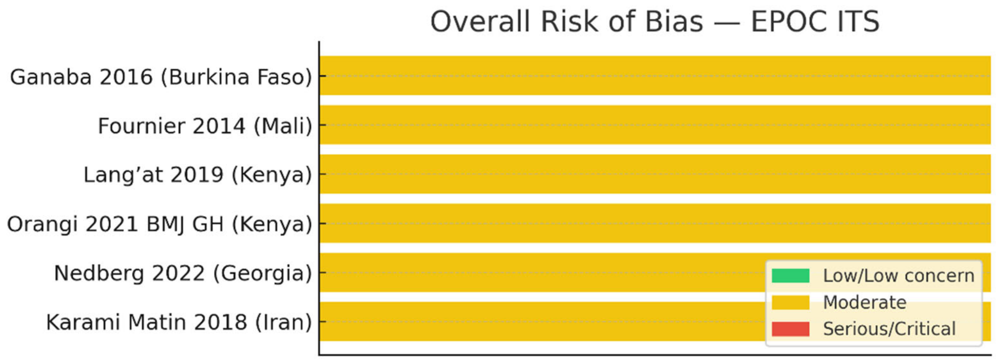


**Figure S1b: EOPC-ITS Overall Traffic Light**

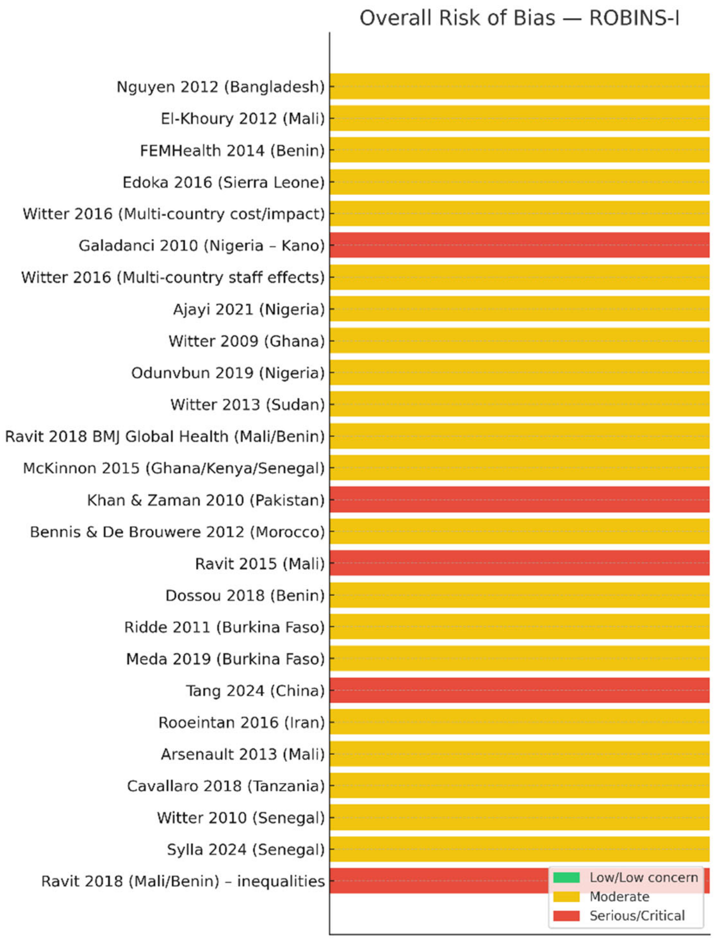


**Figure S1c: ROBIN-I Overall Traffic Light**
